# Three Aphid-Transmitted Viruses Encourage Vector Migration From Infected Common Bean (*Phaseolus vulgaris*) Plants Through a Combination of Volatile and Surface Cues

**DOI:** 10.3389/fpls.2020.613772

**Published:** 2020-12-14

**Authors:** Francis O. Wamonje, Trisna D. Tungadi, Alex M. Murphy, Adrienne E. Pate, Christine Woodcock, John C. Caulfield, J. Musembi Mutuku, Nik J. Cunniffe, Toby J. A. Bruce, Christopher A. Gilligan, John A. Pickett, John P. Carr

**Affiliations:** ^1^Department of Plant Sciences, University of Cambridge, Cambridge, United Kingdom; ^2^Rothamsted Research, Harpenden, United Kingdom; ^3^Biosciences Eastern and Central Africa, International Livestock Research Institute, Nairobi, Kenya

**Keywords:** black bean aphid, green peach aphid, *bean common mosaic virus*, *bean common mosaic necrosis virus*, *cucumber mosaic virus*, non-persistent transmission, virus-induced plant volatiles, electroantennography

## Abstract

Bean common mosaic virus (BCMV), bean common mosaic necrosis virus (BCMNV), and cucumber mosaic virus (CMV) are important pathogens of common bean (*Phaseolus vulgaris*), a crop vital for food security in sub-Saharan Africa. These viruses are vectored by aphids non-persistently, with virions bound loosely to stylet receptors. These viruses also manipulate aphid-mediated transmission by altering host properties. Virus-induced effects on host-aphid interactions were investigated using choice test (migration) assays, olfactometry, and analysis of insect-perceivable volatile organic compounds (VOCs) using gas chromatography (GC)-coupled mass spectrometry, and GC-coupled electroantennography. When allowed to choose freely between infected and uninfected plants, aphids of the legume specialist species *Aphis fabae*, and of the generalist species *Myzus persicae*, were repelled by plants infected with BCMV, BCMNV, or CMV. However, in olfactometer experiments with *A. fabae*, only the VOCs emitted by BCMNV-infected plants repelled aphids. Although BCMV, BCMNV, and CMV each induced distinctive changes in emission of aphid-perceivable volatiles, all three suppressed emission of an attractant sesquiterpene, α-copaene, suggesting these three different viruses promote migration of virus-bearing aphids in a similar fashion.

## Introduction

Two aphid species, *Aphis fabae* (a bean specialist) and *Myzus persicae* (a generalist) have been associated with the spread of viruses in common bean (*Phaseolus vulgaris*) (Worrall et al., 2015). These viruses include the potyviruses bean common mosaic virus (BCMV) and bean common mosaic necrosis virus (BCMNV), and the cucumovirus cucumber mosaic virus (CMV) (Palukaitis and García-Arenal, 2003; Morales, 2006; Jacquemond, 2012). Aphids, including the legume specialist *A. fabae* and the generalist aphid, *M. persicae*, transmit these viruses non-persistently (Morales, 2006; Jacquemond, 2012; Thompson et al., 2015). It was recently shown that on bean plants infected with any of these three viruses, individuals of both aphid species experienced increased feeding difficulties, an effect likely to promote virus transmission (Wamonje et al., 2020). However, there were species-specific differences. Specifically, *A. fabae* feed without difficulty on uninfected plants, whilst *M. persicae* exhibited feeding difficulties, regardless of plant infection status, making bean an unlikely host plant for long term settlement by this generalist aphid (Wamonje et al., 2020).

However, it is not only gustatory cues that determine choice of a host plant by aphids. Host plant selection by aphids is a multi-stage process involving aphid orientation to and identification of a host plant followed by assessment of surface, olfactory and gustatory cues (Bruce, 2015; Mauck et al., 2016). An aphid’s initial choice can be guided through perception of volatile organic compounds (VOCs) emitted by the host plant, but it should be noted that these insects respond to a blend of VOCs rather than to any single compound (Webster et al., 2008, 2010; Bruce and Pickett, 2011).

Some viruses, by altering plant metabolism, particularly by changing emission of VOCs or the accumulation of nutrients or distasteful compounds in plant tissue, can manipulate host-vector interactions in ways that favor their onward transmission (Casteel et al., 2014, 2015; Mauck et al., 2016; Groen et al., 2017; Carr et al., 2018; Donnelly et al., 2019). For example, CMV and potyviral gene products can affect plant VOC emission or accumulation of non-volatile metabolites to stimulate dispersal or settling of aphids on different plant hosts (Lewsey et al., 2010; Ziebell et al., 2011; Westwood et al., 2013; Casteel et al., 2014, 2015; Rhee et al., 2020; Tungadi et al., 2020). In squash and cucumber, infection with CMV increases emission of aphid-attracting VOCs while also causing accumulation of distasteful soluble compounds (Mauck et al., 2010; Carmo-Sousa et al., 2014). These induced changes in VOC emission and the accumulation of distasteful compounds deter aphids from settling, which will accelerate localized virus transmission (Donnelly et al., 2019). Aphid responses also vary between different plant-virus combinations and there can be attraction, repulsion or neutral effects on aphid behavior (Eigenbrode et al., 2002; Jiménez-Martínez et al., 2004; Medina-Ortega et al., 2009; Tungadi et al., 2017).

In work presented here, we investigated how three different viral pathogens of bean (BCMV, BCMNV, and CMV) influence the behavior of specialist and non-specialist aphids by examining the effects of virus infection on aphid host choice, and on emission of aphid-perceivable VOCs. We found that all three viruses exerted similar effects on aphids of both species that are consistent with the encouragement of migration of virus-bearing aphids away from infected plants.

## Materials and Methods

### Viruses, Insects and Plants

Freeze-dried infected leaf tissue for BCMV isolate PV-0915, BCMNV isolate PV-0413 and a bean isolate of CMV (PV-0473) were obtained as from the Deutsche Sammlung von Mikroorganismen und Zellkulturen GmbH (DSMZ) (German Collection of Microorganisms and Cell Cultures). Experiments were conducted with the Red Haricot-GLP 585 cv. “Wairimu” of common bean (*P. vulgaris* L.) (SimLaw Seeds, Nairobi, Kenya). Two aphid (Aphididae: Hemiptera) species, *M. persicae* Sulzer (peach-potato or green peach aphid) and *A. fabae* Scopoli (black bean aphid), were reared on Chinese cabbage (*Brassica rapa* subspecies *pekinensis*) cv. Green Rocket (Tozer Seeds, Cobham, United Kingdom) and on broad bean (*Vicia faba* L.) cv. Sutton (Tozer Seeds), respectively. Bulking of viral inocula, plant growth, virus inoculation, aphid culture, and verification of plant infection by reverse transcription coupled polymerase chain reaction assays with virus-specific primers was described previously (Wamonje et al., 2020).

### Aphid Choice Assays

Two bean seedlings were placed in rectangular pots at the two-leaf stage and 5 days later one plant in each pot was inoculated with virus and the other was mock-inoculated. 10 days later 25 adult apterous (i.e., wingless) aphids of mixed ages were placed in a microfuge tube using a fine brush that was then placed equidistantly between the two plants and the aphids allowed to “choose” to migrate either to the virus-infected or the mock-inoculated plants (Supplementary Figure 1). Aphids that settled on the plants were counted at 1 (*t* = 1 h) and 24 (*t* = 24 h) hours post-placement. For each virus treatment, at least 15 replicates were done. ‘Normal illumination’ (16 h light/8h dark) and continuous dark conditions used in this study were the same as those described by Tungadi et al. (2017).

### Entrainment and Analysis of Headspace VOCs

Collection of headspace VOCs by air entrainment from either virus-infected or mock-inoculated plants was done over a 5-day period as previously described (Beale et al., 2006; Groen et al., 2016; Tungadi et al., 2017). Four samples (each from a single plant) were collected per treatment. Briefly, VOCs were separated on a capillary gas chromatography (GC) column [50 m × 0.32 mm i.d., 0.52 μm film thickness, ZB-1, Zebron (Phenomenex, Torrance, CA, United States)] fitted with a programmable temperature vaporizer. The GC was directly coupled to a mass spectrometer (DSQ II, Thermo Scientific, Bremen, Germany). Ions were detected between 30 and 650 m/z (mass/charge ratio). Data was collected using Xcalibur software (Thermo Scientific). Principal component analysis (PCA) of mass spectra was performed with MetaboAnalyst 2.0 (Xia et al., 2012), as previously described (Groen et al., 2016).

For identification of VOCs, the four samples in each treatment group were pooled into one vial and then concentrated down to 10 μl to improve detection by blowing a steady stream of nitrogen into the vial. A 2 μl aliquot of the sample was injected onto a capillary GC column (50 m × 0.32 mm i.d., HP-1) directly coupled to a mass spectrometer (Thermo Finnigan, MAT95, Bremen, Germany) under conditions previously described (Groen et al., 2016). Compounds were tentatively identified by comparing spectra with those in the National Institute of Standards and Technology (NIST) spectral databases^1^. Confirmation of identity was by use of authentic commercially available samples in the GC including measuring the Kováts indices for comparison with the recorded NIST values. The chiral sesquiterpene α-copaene was identified using the (-) isomer, the most common isomer found *in planta*. For identification of electrophysiologically active VOCs, i.e., VOCs that evoke responses in aphid antennal preparations, coupled gas chromatography–electroantennography (GC-EAG) recordings for *A. fabae* were carried out as previously described (Webster et al., 2008; Sobhy et al., 2017). VOCs were separated on an HP-1 column (50 m × 0.32-mm i.d.) in a Hewlett-Packard 6890 gas chromatograph (Agilent Technologies, Santa Clara, CA, United States) equipped with a cold on-column injector and a flame ionization detector (FID). Electroantennogram signals were passed through a high-impedance amplifier (UN-06: Syntech, Netherlands), and simultaneous recordings of the EAG and FID responses were obtained using specialized software (EAD version 2.3: Syntech).

### Olfactometry Experiments

The olfactometer design was adapted from that devised by Safari et al. (2019). The two bean plants providing the volatiles were placed under separate bell jars (300 mm tall with an internal diameter of 100 mm). A layer of fine muslin mesh was placed within the bell jar and above the plant to allow the volatiles to diffuse through without permitting the aphids to make physical contact with the bean plants. A plastic box measuring 170 mm × 115 mm × 50 mm (length × width × depth) was placed on top of these jars, lid side down. The box lid in contact with the top of the bell jars had two holes that matched the dimensions of the pipe-like protrusion at the top of each bell jar and a third hole equidistant between the other two which was used to hold a 1.5 ml microfuge tube containing the aphids. The plastic box was covered with aluminum foil was used to keep aphids in darkness to remove visual cues. An open microfuge tube containing 100 7-day-old wingless aphids was placed in the hole in the middle of the box after an hour of starvation at 22°C and the choices recorded after 24 h.

### Statistical Analyses

A Bayesian approach based on fitting binomial probability distributions to choice test data was used to assess whether aphids preferentially settled on infected plants. Data were modeled from individual pots in a single choice test as independent samples with a probability of “success” (i.e., choosing an infected plant) fixed for each experiment at each time as previously described (Tungadi et al., 2017). The probability that aphids would settle on an infected plant was estimated at 1 (*p*_1_) and 24 h (*p*_24_) post-placement, and the level of support from the data for any preference in aphid choices assessed by checking whether or not the 95% credible intervals for *p*_1_ and *p*_24_ overlapped 0.5. The Chi-square (χ^2^) “goodness-of-fit” test was used for the olfactometry experiments.

## Results

### Aphids Preferentially Settle on Uninfected Plants

**FIGURE 1:**
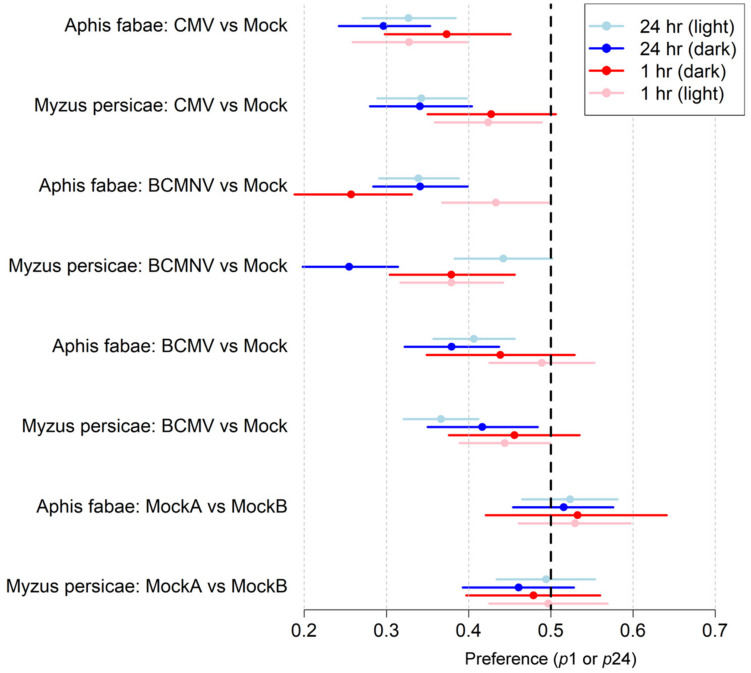
The probability of aphids choosing to settle on mock-inoculated plants is higher than on virus-infected plants. Summary of the data obtained from aphid free choice assays performed under normal illumination and in darkness (Tables 1, 2). Data is expressed so that a lower probability value for settling on virus-infected plants indicates a higher preference for aphids to settle on mock-inoculated plants (Tungadi et al., 2017). Twenty-five wingless aphids were placed equidistantly between a mock-inoculated plant and a virus-infected plant [inoculated with either cucumber mosaic virus (CMV), bean common mosaic virus (BCMV), or bean common mosaic necrosis virus (BCMNV)]. Individuals of both aphid species (*A. fabae* and *M. persicae*) preferred to settle on uninfected plants both under normal illumination (Table 1) and in darkness (Table 2) after 1 and 24 h. All the credible intervals were statistically significant for all virus-infected vs. mock-inoculated plant combinations except for *M. persicae* on BCMNV-infected plants under normal illumination (95% credible interval 0.38–0.050). Aphids did not distinguish between two mock-inoculated plants at either 1- or 24-h either under normal illumination or in continuous darkness. The *X*-axis shows the range of the 95% credible interval of the parameters, *p*1 and *p*24 that are the probabilities of choosing to settle on an infected plant conditional on settling at *t* = 1 h and *t* = 24 h, respectively. The colored dots represent the mean, and the colored lines represent the credible interval. The dotted vertical black line represents equal probability.

Under normal lighting conditions, aphids of both species (*A. fabae* and *M. persicae*) preferred to settle on mock-inoculated rather than virus-infected bean plants (Table 1 and Figure 1). By 1 h following release aphids of both species had settled preferentially on mock-inoculated plants. For all three viruses, the 95% credible interval for the probability of selecting an infected plant did not overlap 0.5. In particular, for *A. fabae* choosing between CMV-infected and mock-inoculated plants (95% credible interval: 0.26–0.40), for *M. persicae* choosing between CMV-infected vs. mock-inoculated plants (95% credible interval: 0.36–0.49), and BCMNV vs. mock-inoculated plants (95% credible interval: 0.32–0.44). The same preference of aphids for mock-inoculated plants was also seen at 24 h (Table 1 and Figure 1).

**TABLE 1:** Analysis of aphid settling choices in different choice test combinations under normal illumination.

Choice Test combinations	*Aphis fabae*						*Myzus persicae*					
	1 h (*p*_1_)			24 h (*p*_24_)			1 h (*p*_1_)			24 h (*p*_24_)		
	Responding aphids (15 Reps, *N* = 375)			Responding aphids (15 Reps, *N* = 375)			Responding aphids (15 Reps, *N* = 375)			Responding aphids (15 Reps, *N* = 375)		
	Infected	Mock	95% Credible interval	Infected	Mock	95% Credible interval	Infected	Mock	95% Credible interval	Infected	Mock	95% Credible interval
CMV-MOCK	55	114	0.26–0.40*	84	174	0.27–0.38*	88	115	0.36–0.49*	94	173	0.29–0.40*
BCMV-MOCK	112	117	0.42–0.55	151	221	0.36–0.46*	134	168	0.39–0.50	151	262	0.32–0.41*
BCMNV-MOCK	93	122	0.37–0.50	121	237	0.29–0.39*	85	140	0.32–0.44*	114	144	0.38–0.50
MOCK-MOCK	94	106	0.46–0.60	130	143	0.46–0.58	75	81	0.42–0.57	113	120	0.43–0.55

Asterisk (*) denotes where 95% confidence intervals did not overlap 0.5.

In darkness, aphids of both species also settled preferentially on uninfected plants. By 1 h after release, *A. fabae* were more likely to settle on mock-inoculated than CMV-infected (95% credible interval: 0.30–0.45), or on BCMNV-infected plants (95% credible interval: 0.19–0.33). Similarly, *M. persicae* chose to settle on mock-inoculated plants in preference to BCMNV-infected plants (95% credible interval: 0.30–0.46) (Table 2 and Figure 1). By 24 h, in all combinations where virus-infected plants were present, the data supported a preference for mock-inoculated plants (Table 2 and Figure 1). Neither *A. fabae* nor *M. persicae* showed any preference when presented with pairs of mock-inoculated plants either under normal illumination conditions or in darkness.

**TABLE 2:** Analysis of aphid settling choices in different choice test combinations in darkness.

Choice Test combinations	*Aphis fabae*						*Myzus persicae*					
	1 h (*p*_1_)			24 h (*p*_24_)			1 h (*p*_1_)			24 h (*p*_24_)		
	Responding aphids (15 Reps, *N* = 375)			Responding aphids (15 Reps, *N* = 375)			Responding aphids (15 Reps, *N* = 375)			Responding aphids (15 Reps, *N* = 375)		
	Infected	Mock	95% Credible interval	Infected	Mock	95% Credible interval	Infected	Mock	95% Credible interval	Infected	Mock	95% Credible interval
CMV-MOCK	55	114	0.30–0.45*	84	174	0.24–0.35*	64	86	0.35–0.54	74	144	0.28–0.40*
BCMV-MOCK	49	63	0.35–0.53	101	166	0.32–0.44*	66	79	0.38–0.54	84	118	0.35–0.48*
BCMNV-MOCK	35	103	0.19–0.33*	86	167	0.28–0.40*	58	95	0.30–0.46*	59	162	0.20–0.31*
MOCK-MOCK	35	40	0.42–0.58	123	131	0.45–0.58	73	67	0.40–0.56	109	93	0.39–0.53

Asterisk (*) denotes where differences did not overlap 0.5.

### Changes in Plant VOC Emission Engendered by BCMNV Were Sufficient to Influence Aphid Preference for Uninfected Plants

**FIGURE 2:**
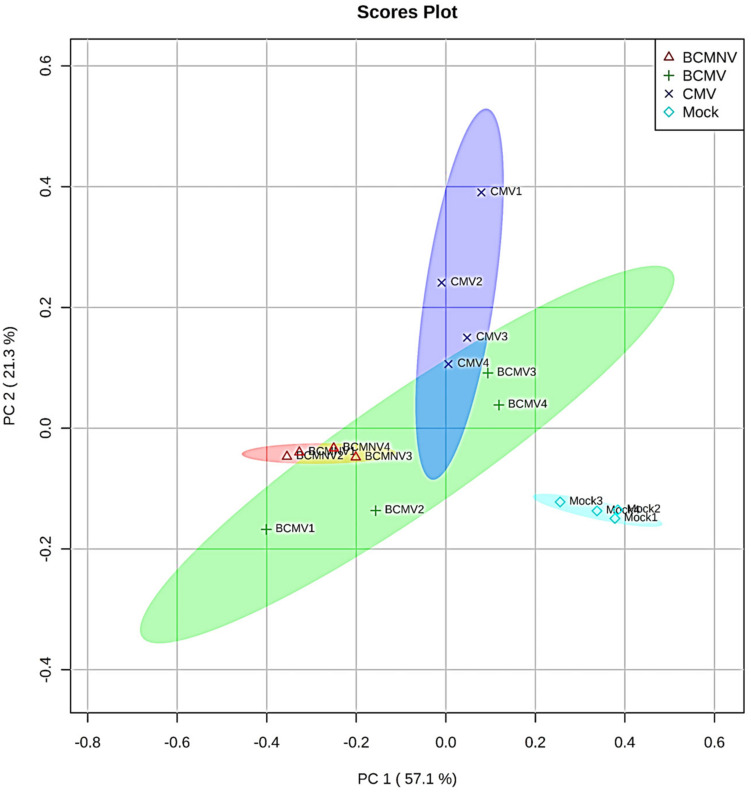
Principal component analysis (PCA) of mass spectrometry data shows qualitative differences in volatile organic compound (VOC) blends. A score scatter plot of PCA of the m/z values (binned to 1.0 Da) obtained by gas chromatography-mass spectrometry of samples of VOCs collected by dynamic headspace trapping from bean plants. Samples collected from four plants in each treatment group; mock-inoculated (light blue color) BCMV (green), BCMNV (pink), and CMV (dark blue) infected plants were compared. The analysis showed discrimination between mock-inoculated and virus-infected plants and revealed an overlap between the principal ions representing the VOC blends from the virus treatments. The percentage of variation of the data explained by PC1 and PC2 is in parentheses (57.1 and 21.3%, respectively).

**FIGURE 3:**
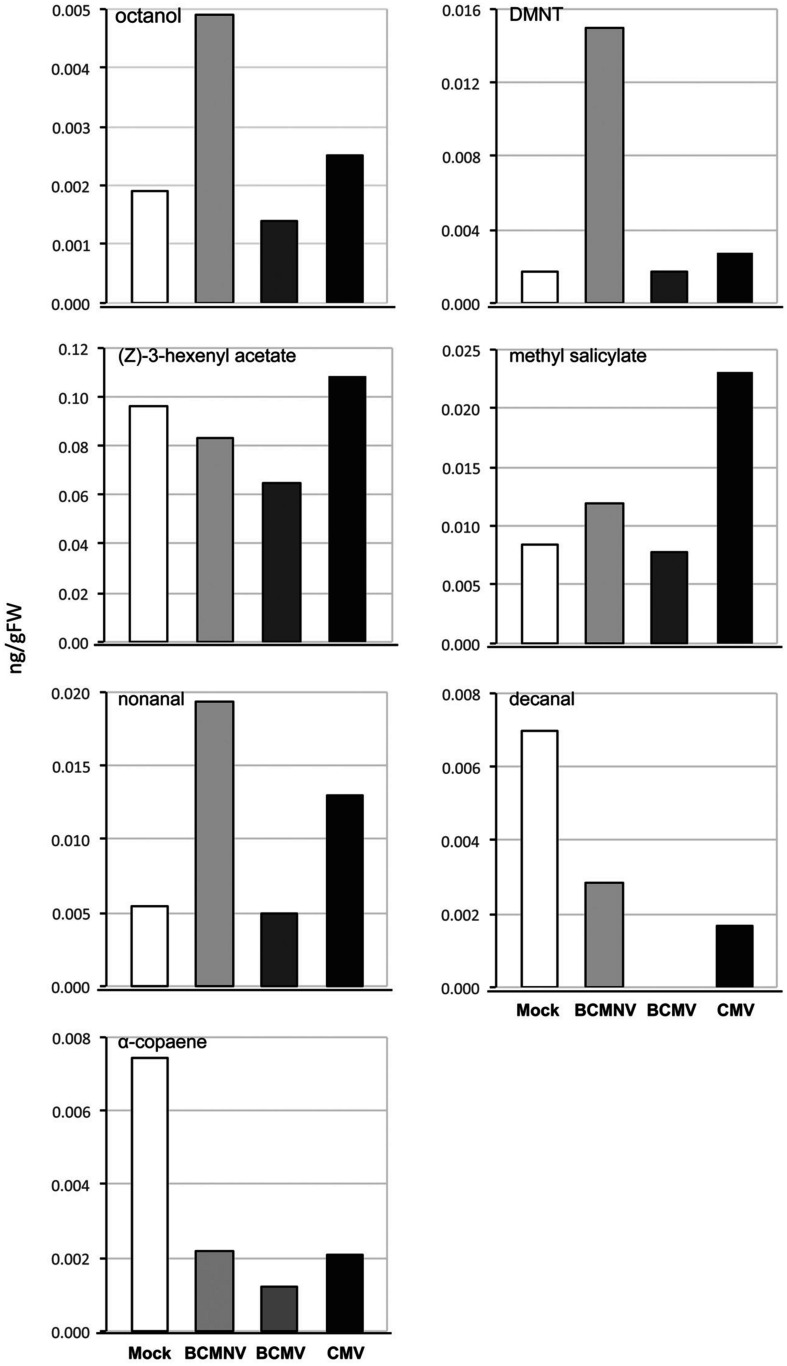
Virus infection modifies emission of electrophysiologically active volatile organic compounds (VOCs) by bean plants. Virus infection engendered differences in the production levels of seven electrophysiologically active VOCs. Notably, α-copaene levels were diminished in VOC blends emitted by bean plants infected with bean common mosaic virus (BCMV), bean common mosaic necrosis virus (BCMNV), and cucumber mosaic virus (CMV). Decanal levels were also diminished by infection and the VOC was below the limits of detection of samples from headspace of BCMV-infected plants. Other VOC levels varied across different treatments. CMV and BCMNV infection appeared to elicit the most marked changes in levels of electrophysiologically active compounds. VOCs from CMV-infected plants had elevated methyl salicylate, nonanal, octanal and (*Z*)-3-hexenyl acetate levels. Uniquely, BCMNV-infected plants had elevated (*E*)-8-dimethyl-1,3,7- nonatriene (DMNT), octanol and nonanal levels. The data shows VOCs expressed as ng/gFW of bean plants collected over 5 days by dynamic headspace VOC collection and analyzed by GC-MS.

In the olfactometry tests conducted at 10 days post-inoculation, significantly more aphids preferred uninfected plants to BCMNV-infected plants (Table 3). The other virus vs. mock combinations and mock vs. mock combinations did not produce significant differences (Table 3). This indicates that there are differences in the VOC blend conditioned by different viruses. Also, that the overall influence on aphid choices exerted through VOCs may vary from virus to virus and that other cues have a role in determining eventual choice of host plant for long-term settling. Qualitative and quantitative changes in the VOC blends emitted from bean plants in response to virus infection were revealed by GC-MS analysis. PCA demonstrated overall qualitative differences in the VOC blends of virus-infected plants (Figure 2). The blend from mock-inoculated plants clustered separately from the VOC blends emitted by the virus-infected plants and the PCA indicated there were overall much greater similarities in the blends emitted by plants infected with BCMV, BCMNV, and CMV than between infected and uninfected (mock-inoculated) plants (Figure 2). Several VOCs were identified as electrophysiologically active by GC-EAG. The electrophysiological responses triggered by these compounds indicate that they are insect-perceivable and are potential olfactory cues that may help aphids identify or distinguish between virus-infected and uninfected plants (Figure 3 and Supplementary Figure 2). Virus infection affected emission of these electrophysiologically active VOCs in different ways (Figure 3). The abundance of (*Z*)-3-hexenyl acetate was unaffected by infection status of the plant. Nonanal and octanal were elevated in blends produced by BCMV- and CMV-infected plants. (*E*)-4,8-dimethyl-1,3,7-nonatriene (DMNT) was elevated in the blend emitted by BCMNV-infected plants. Methyl salicylate was elevated in blends emitted by CMV-infected plants (Figure 3). Decanal (which was below the limit of detection in headspace samples of BCMV-infected plants) and α-copaene were less abundant in blends emitted by infected plants (Figure 3).

**TABLE 3:** Olfactometry test with *Aphis fabae* over 24 h.

Responding aphids (7 Reps, *N* = 700) MOCK A vs. MOCK B		Responding aphids (7 Reps, *N* = 700) MOCK vs. BCMV		Responding aphids (7 Reps, *N* = 700) MOCK vs. CMV		Responding aphids (7 Reps, *N* = 700) MOCK vs. BCMNV	
25	43	12	24	14	50	44	30
59	14	21	15	50	35	49	23
22	39	18	41	7	31	59	48
44	37	50	8	48	13	48	9
29	49	37	32	15	43	47	21
45	27	23	55	34	7	57	33
19	23	15	16	26	55	50	11
243	232	176	191	194	234	354	175

χ2 (df 1, *n* = 475) = test statistic, 0.254736842, *p* = 0.61		χ2 (df 1, *n* = 385) = test statistic, 0.023376623, *p* = 0.88		χ2 (df 1, *n* = 428) = test statistic, 3.738317757, *p* = 0.053		χ2 (df 1, *n* = 529) = test statistic, 60.56871672, *p* = 7.10 × 10^–15^*	

Asterisk (*) denotes where differences were highly statistically significant.

## Discussion

Aphids of a bean specialist species (*A. fabae*) and of a generalist species (*M. persicae*) prefer to settle on uninfected bean plants rather than on plants infected with BCMV, BCMNV, or CMV. We concluded from free choice settling experiments under normal illumination and in darkness that volatile cues play a key role in conditioning these choices. Our GC-MS analyses of plant VOCs confirmed that all three viruses induce qualitative changes in the profile of emitted metabolites, including VOCs that were electrophysiologically active in EAG assays.

Perception of VOCs provides odor cues that contribute to initial host choices and the location of the host plant (Bruce and Pickett, 2011). Subsequently, aphids use antennal chemosensory hairs and stylet probes to assess surface and taste cues to determine whether the leaf surface is suitable (Powell et al., 1995). We hypothesized that the viruses could influence these plant cues and thereby affect the outcome of the aphids’ host selection process. In all free choice experiments, more aphids settled on mock-inoculated plants than on virus-infected plants within an hour. All three viruses induce changes in emission of electrophysiologically active VOCs that may help to explain migration away from infected plants and toward uninfected plants: an effect that would promote local virus transmission (Donnelly et al., 2019).

Data from free choice experiments (when aphids were able to settle on plants) and olfactometry experiments (where the aphids could not access the plants) enabled us to deduce a role for surface cues. Only BCMNV-infected plants had a sufficiently strong olfactometry signal to influence aphid choices (in favor of uninfected plants). Conversely, when aphids could access a plant to settle on, there was a preference for uninfected plants by 24 h in all the virus treatments. This is strongly suggestive of a role of surface cues in addition to gustatory cues, shown in our earlier work (Wamonje et al., 2020).

### Changes to VOCs Suggest That Virus Infection May Affect Host Plant Location by Aphids

Aphids use blends of VOCs rather than individual semiochemicals to identify plants as hosts, as shown by *A. fabae* when locating *V. faba* plants (Webster et al., 2008). It follows that the virus-induced changes in the VOC blends we observed might affect the ability of aphids to locate host plants, although it should be noted that this is not always the case, as shown previously with CMV-infected tobacco (Tungadi et al., 2017). Seven electrophysiologically active VOCs were identified by GC-EAG that were consistently found in VOC blends emitted by bean plants. Though this discussion is limited to the possible role some of these VOCs could play, it is not implied that these are the only components of the emitted blends that might affect aphid behavior.

Comparisons of individual treatments showed that the proportion of (*Z*)-3-hexenyl acetate in VOC blends varied, being lowest for BCMV-infected and highest for CMV-infected bean plants. This may have contributed to the virus-induced changes in VOC emission revealed by PCA analysis. (*Z*)-3-hexenyl acetate has a role in location of host plants by *A. fabae* (Webster et al., 2008), and causes attraction of large raspberry aphids (*Amphorophora idaei*) to strawberries (*Rubus idaeus*) infected with black raspberry necrosis virus or raspberry leaf mottle virus (McMenemy et al., 2012). While it was not in the scope of this set of experiments to test the role of this compound, we can speculate that this VOC plays a role in the aphid selection of virus-infected plants.

Another VOC with an established role in aphid host location is methyl salicylate. For example, it has been shown that methyl salicylate masks the location of favorable host plants from *A. fabae* (Hardie et al., 1994). We detected a higher proportion of methyl salicylate in the VOC blend emitted by CMV-infected plants. Thus, it is possible that the decreased settling of aphids observed particularly on CMV-infected bean plants may be partly attributed to increased methyl salicylate emission but it is unlikely to explain the repellence of plants infected with BCMV or BCMNV. Similarly, DMNT can sabotage host plant location by aphids by suppressing perception of plant odor (Hatano et al., 2015), and its emission may help explain the repellence of BCMNV-infected plants but it is unlikely to be a major influence on aphid repellence by BCMNV-infected or CMV-infected plants. Infection by all three viruses consistently suppressed the emission of decanal and α-copaene. Decanal can aid in host location for *A. fabae*, but only when it is a component of a VOC blend; the pure compound is repellent (Webster et al., 2010). Emission of α-copaene is induced by aphid infestation and it also appears to be an aphid-attracting VOC (Stewart-Jones and Poppy, 2006; Saad et al., 2015; Clancy et al., 2016; Sobhy et al., 2017). The decrease in α-copaene emission that we saw consistently in bean plants infected by all three of these viruses, and perhaps also the consistent decrease in decanal emission, suggests a possible common mechanism of discouragement of aphid settlement on virus-infected plants. Specifically, that these three viruses, two of which are legume specialists and one (CMV) that has one of the widest host ranges (Worrall et al., 2015; Yoon et al., 2019), have acquired the same “strategy” for encouraging the dispersal of virus-bearing aphids. That is, by decreasing the complexity of the plant blend, and in particular by down-regulating emission of more attractive VOCs, these viruses make plants less hospitable-seeming or less salient than uninfected plants, rather than by making them more repellent.

## Conclusion

Three agronomically important common bean viruses induce alterations in VOC emission that are likely to cause changes in aphid-host interactions and promote dispersal of aphids. Several authors have suggested that such virus-induced changes increase the likelihood of aphid-mediated virus transmission to adjacent uninfected host plants (Carmo-Sousa et al., 2014; Mauck et al., 2016; Groen et al., 2017; Wamonje et al., 2020). However, recent theoretical analyses indicate that virus-driven enhancement of aphid dispersal might be self-limiting for non-persistently transmitted viruses due to consequent decreases in aphid population density (Carr et al., 2018; Donnelly et al., 2019). It will, therefore, be important to see how these virus-induced changes in plant volatile emission affect host-vector interactions and subsequent transmission of BCMV, BCMNV, and CMV under field conditions. Better understanding of how VOCs and other cues affect virus transmission could inform new methods to inhibit aphid-mediated virus transmission (Bruce et al., 2015). Similar insights have led to successful mixed cropping systems that inhibit infestation by lepidopteran pests (Pickett and Khan, 2016). Additionally, development of sensors for virus-specific changes in plant VOC blends may offer a new avenue for detection and monitoring of virus infection in the field.

## Data Availability Statement

The original contributions presented in the study are included in the article/Supplementary Material, further inquiries can be directed to the corresponding author/s.

## Author Contributions

JPC, FW, JP, TB, and CG planned and designed the research. FW, TT, AM, AP, CW, JC, and JM performed the experiments. FW, NC, CG, TB, JP, AM, and JPC analyzed the data. FW, AM, and JPC wrote the manuscript with contributions from all authors.

## Conflict of Interest

The authors declare that the research was conducted in the absence of any commercial or financial relationships that could be construed as a potential conflict of interest.

## Footnotes

1 http://www.nist.gov

## Supplementary Material

Supplementary Figure 1 Experimental set up for aphid free-choice tests.

Supplementary Figure 2 Coupled gas chromatography–electroantennography (GC-EAG).
