# Supplementary material for: Three Aphid-Transmitted Viruses Encourage Vector Migration From Infected Common Bean (Phaseolus vulgaris) Plants Through a Combination of Volatile and Surface Cues
Source: Front Plant Sci. 2020 Dec 14;11:613772. doi: 10.3389/fpls.2020.613772 (PMC7767818; doi:10.3389/fpls.2020.613772)
Supplement: Supplementary Figure 1 — Experimental set up for aphid free-choice tests. [file Data_Sheet_1.PDF]

Supplementary Material

Three aphid-transmitted viruses encourage vector migration from infected common bean (*Phaseolus vulgaris*) plants through a combination of volatile and surface cues

Francis O. Wamonje[1♮], Trisna D. Tungadi[1δ], Alex M. Murphy[1], Adrienne E. Pate[1], Christine Woodcock[2], John Caulfield[2], J. Musembi Mutuku[1,3ϕ], Nik J. Cunniffe[1], Toby J. A. Bruce[2*], Christopher A. Gilligan[1], John A. Pickett[2§] and John P. Carr[1#].

1. Department of Plant Sciences, University of Cambridge, Cambridge CB2 3EA, UK

2. Rothamsted Research, Harpenden, Hertfordshire AL5 2JQ, UK

3. Biosciences eastern and central Africa, International Livestock Research Institute, P.O. Box 30709-00100, Nairobi, Kenya.

[#] Corresponding author: jpc1005@hermes.cam.ac.uk

♮ Present address: International Centre of Insect Physiology and Ecology, 30772-00100 Nairobi, Kenya

δ Present address: Pest and Pathogen Ecology, NIAB EMR, East Malling, Kent, UK ME19 6BJ

*Present address: School of Life Sciences, Keele University, Staffordshire ST5 5BG, UK

§ Present address: School of Chemistry, Cardiff University, Cardiff, CF10 3AT, UK

[ϕ] Present address: The Central and West Africa Virus Epidemiology (WAVE) program, Université Felix Houphouët Boigny, Cote d'Ivoire.

Supplementary Figures

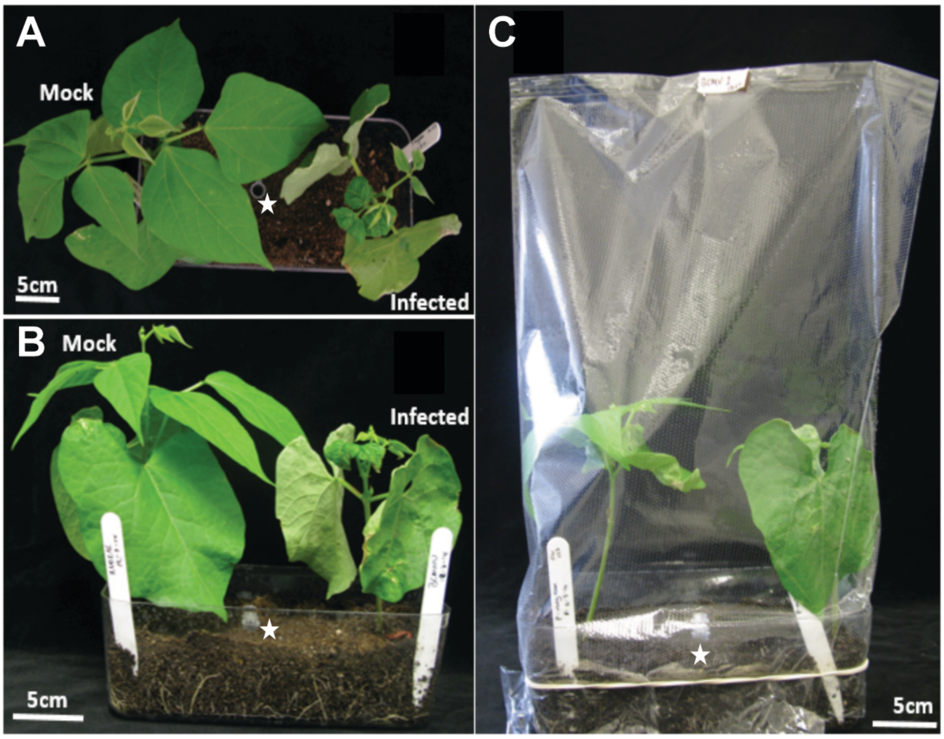

SUPPLEMENTARY FIGURE 1. Experimental set up for aphid free-choice tests. In this example the bean plant labeled ‘Infected’ had been inoculated with BCMNV 10 days previously at the two-leaf stage, while sterile water had been used for mock-inoculation (plant labeled ‘Mock’). Panels **A** and **B** show, respectively, views from above and from the side of the experimental set up. Aphids (*Myzus persicae* or *Aphis fabae*) were released from a microfuge tube placed equidistantly between the plants (indicated by a white star) and their settling behavior observed for up to 24 h post-release. A micro-perforated bag, which is porous to air but which confines aphids, was secured around the set up so as to prevent the aphids from escaping **(C)**.

A Typical GC-EAG readout for headspace VOCs

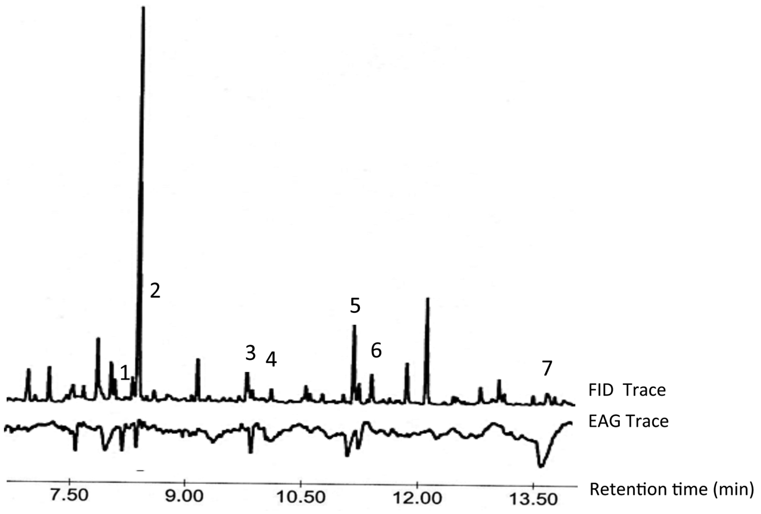

B Key to electrophysiologically-active VOCs detected

Peak Number	KI in GC-EAG	Exp KI	VOC ID	Comments
1	983	982	octanal	Highest levels emitted by BCMNV-infected plants
2	987	988	(*Z*)-3-hexenyl acetate	Abundant in all treatments
3	1084	1084	nonanal	Higher levels emitted by virus-infected plants
4	1106	1106	DMNT	Highest levels emitted by BCMNV-infected plants
5	1177	1173	methyl salicylate	Highest levels emitted by CMV-infected plants
6	1186	1185	decanal	Lower levels emitted by plants infected with BCMV, BCMNV or CMV.
7	1375	1381	α-copaene	Lower levels emitted by plants infected with BCMV, BCMNV or CMV.

SUPPLEMENTARY FIGURE 2. Coupled gas chromatography–electroantennography (GC-EAG). Example of an amalgamated GC-EAG recording for an *A. fabae* antennal preparation responding to headspace volatile organic compounds (VOCs) emitted by CMV-infected bean plants (**a**). The upper trace represents the flame ionization detector response following GC and the lower trace represents the aphid electroantennography (EAG) response. The key (**b**) indicates the electrophysiologically active VOC peaks, 1-7, as well as the actual and expected Kovats retention index (KI) for these VOCs.
